# Collective route memories emerge through differential forgetting of navigational information in homing pigeons

**DOI:** 10.1038/s41598-026-39898-2

**Published:** 2026-03-08

**Authors:** Joe Morford, Patrick J. Lewin, Richard P. Mann, Christopher Krupenye, Dora Biro

**Affiliations:** 1 Department of Brain and Cognitive Sciences, University of Rochester, Rochester, NY USA; 2 Department of Biology, Oxford University, Oxford, UK; 3 Department of Statistics, School of Mathematics, University of Leeds, Leeds, UK; 4 Department of Psychological & Brain Sciences, Johns Hopkins University, Baltimore, MD USA

**Keywords:** Ecology, Ecology, Neuroscience, Zoology

## Abstract

Better decision-making in larger groups than smaller groups or individuals has been observed across various taxa. While this phenomenon is thought to result from the pooling of independent information in collective decision-making, an alternative mechanism is the better retention of learned information in larger groups: collective memory. We investigated the emergence of collective memory and its role in collective intelligence by training homing pigeons to navigate home in pairs and testing their retention of learned routes. In a treatment with an eight-week forgetting period between training and memory testing, pairs flew closer to their learned routes than solo-tested birds, likely through differential retention of information across pairs. However, better memory retention in pairs did not translate into better homing efficiency, perhaps because the forgetting period was too short to generate a sufficient drop in efficiency. A second treatment demonstrated that extra training and a shorter forgetting period abolished the difference between paired and solo memory performance. These findings demonstrate that differential retention of information across group members can lead to the emergence of collective memory in animals. This has implications for a wide range of contexts in which the interplay of learning and memory shape individual and collective behaviour.

## Introduction

The phenomenon in which larger groups make better decisions than smaller groups or individuals, a manifestation of collective intelligence, has been observed across various animal taxa^1^. It is typically understood as arising from the pooling of independent information in collective decision-making processes, which improves group-level decision-making through mechanisms like the many-wrongs principle^2^ or Condorcet’s jury theorem^3^. However, there has been little research on the role of memory in collective intelligence^4^. Here, we focus on two issues. First, we examine whether larger groups might demonstrate better memory of learned solutions than smaller groups; we term the better retention of learned solutions in larger groups: ‘collective memory’. Second, we examine whether the better retention of learned solutions in larger groups leads to larger groups exhibiting *better* solutions than smaller groups, i.e., whether collective memory translates into collective intelligence.

Collective memory has been examined experimentally in humans, where findings indicate that collaborative groups – i.e., those tasked with solving a problem together – recall more information than individuals^5–7^. However, it remains unclear whether these effects are dependent on the complex cognitive and communicative abilities of humans (allowing, for instance, cultural transmission of shared ideas or coordination between group members in who remembers what) or if they could emerge in non-human animals. Collective memory could arise if individuals with better retention of memorised solutions have greater input into collective decision-making. In this scenario, the collectively recalled solution would outperform the recalled solution of some individuals (those more likely to act as ‘followers’ in collective decision-making, but not others (leaders). This would provide a performance benefit of group membership (a ‘collective membership gain’, as in^8^ only for followers, and could potentially be costly for leaders if individuals with poorer memory retention also have some control of collective decisions. Alternatively, if individuals remember different parts of the solution, groups might form a distributed memory system and collective memory could emerge through information pooling across individuals. In this scenario, the group would perform closer to the original solution than could any member alone, so all individuals would gain a performance benefit from group membership.

A key question regarding collective intelligence is how members of the same animal group contribute sufficiently diverse information to generate collective intelligence; if group members do not contribute independent information to collective decision-making, perhaps because they have correlated information about the environment, then collective intelligence is not expected to emerge^9–11^. One context in which animals might be able to contribute independent information to collective decision-making is if they learn tasks separately, coming to independent solutions that can be combined, generating collective intelligence (e.g.,^12^. This has been demonstrated in the routes of homing pigeons (*Columba livia*^13^, with larger flocks homing more efficiently than smaller flocks, and smaller flocks more efficiently than solo birds, after individual learning. However, in stable groups, it is unclear whether there would be sufficient opportunity for isolated individual learning to enable the emergence of collective intelligence through this mechanism. Modelling work^10^ has shown how collective intelligence can emerge through collective learning (learning through joint action; see also^14^ in stable groups. This does not require coordination between group members in what they learn, but does depend on the availability of cues with low observational correlation between group members; whether such low-correlation cues are available to be utilised by animals is an open question. Indeed, available empirical evidence suggests that collective learning in stable groups may not always be sufficient for the emergence of collective intelligence. For instance, improvements in route efficiency and fidelity appear to progress at similar rates in both individual homing pigeons and pairs of homing pigeons^15,16^.

An alternative mechanism that might facilitate the emergence of collective intelligence comprises collective learning followed by the differential retention of information between group members. According to this hypothesis, collective intelligence could emerge as a long-term consequence of collective learning, even if it does not manifest immediately during or directly after learning. This relies on the idea that group members retain different relevant cues, with low correlation between group members in the probability of retaining each cue. Such differential retention could create a distributed memory system across the group, allowing the collective to retain a more accurate route memory than any individual member. Moreover, this might subsequently facilitate novel recombination of partial route memories, stimulating further increases in collective performance.

Collective navigation in homing pigeons provides an ideal model for studying collective cognition, as all individuals share the common goal of reaching a home loft, and task performance can be precisely quantified using GPS tracking. This has enabled homing pigeon navigation to be utilised as a model system of collective cognition in recent years, with experiments investigating the emergence of cumulative culture^16^ and collective intelligence in this system^13^. Additionally, pigeons’ ability to develop idiosyncratic homing routes through learning^17,18^ makes them well-suited for memory research, with a recent study demonstrating that pigeons retain partial route memories several years following learning^19^.

**Fig. 1:**
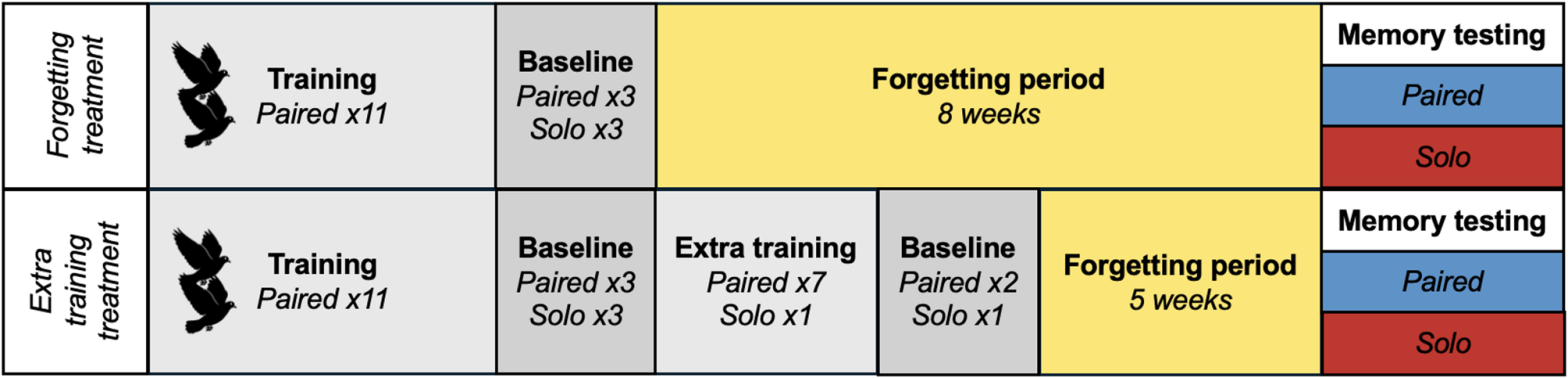
Experimental design. Pairs of homing pigeons completed both treatments, with the site at which they completed each treatment randomly assigned between the two experimental release sites. At the end of training, the final three paired releases were interspersed with three solo releases, all of which were recorded with GPS as baseline routes. Likewise, at the end of extra training, the final two paired releases and a solo release were recorded as baseline routes. At the end of the experiment, a single memory testing release took place either in pairs or solo (randomly assigned). See Methods for full details of experimental design.

Here, we investigated whether collective memory emerges in co-navigating groups of homing pigeons and, if so, whether this results in collective intelligence. We released homing pigeons in stable pairs 14 times, from each of two release sites, to entrain routes from each site back to their home loft. Subsequently, after a several-week period of forgetting, route memory and navigational efficiency were tested in pairs, and in solo pigeons, split from their partners for testing, to assess the emergence of collective memory and collective intelligence. This was implemented with two different treatments to induce differences in the extent to which the pigeons would forget the entrained routes: (1) a forgetting treatment, at one site (chosen randomly for each pair), where the pigeons were not released after the end of training for eight weeks until memory testing; (2) an extra training treatment, at the other site, where pigeon pairs received extra training (nine extra paired releases over three weeks), and a shorter period of forgetting (approximately five weeks) before memory testing (see design in Fig. 1). Route memory was quantified using two metrics to ensure results were robust to different measures: (1) the mean nearest neighbour distance across the route to any of its previously recorded baseline routes from paired releases at the end of training (mean NND); (2) the second order mean of the mean nearest neighbour distances across the route to each of its previously recorded baseline routes from paired releases at the end of training (2nd order mean NND). We report results on the influence and interaction of release condition (pair vs. solo), testing time (baseline testing vs. at memory testing), and treatment (forgetting vs. extra training) on route memory and homing efficiency from linear multimember mixed-effect models (see Methods for details). For each metric (mean NND, 2nd order mean NND, homing efficiency index), fixed effects were tested using single fitted models, with reference levels adjusted to obtain planned contrasts.

## Results and discussion

**Fig. 2:**
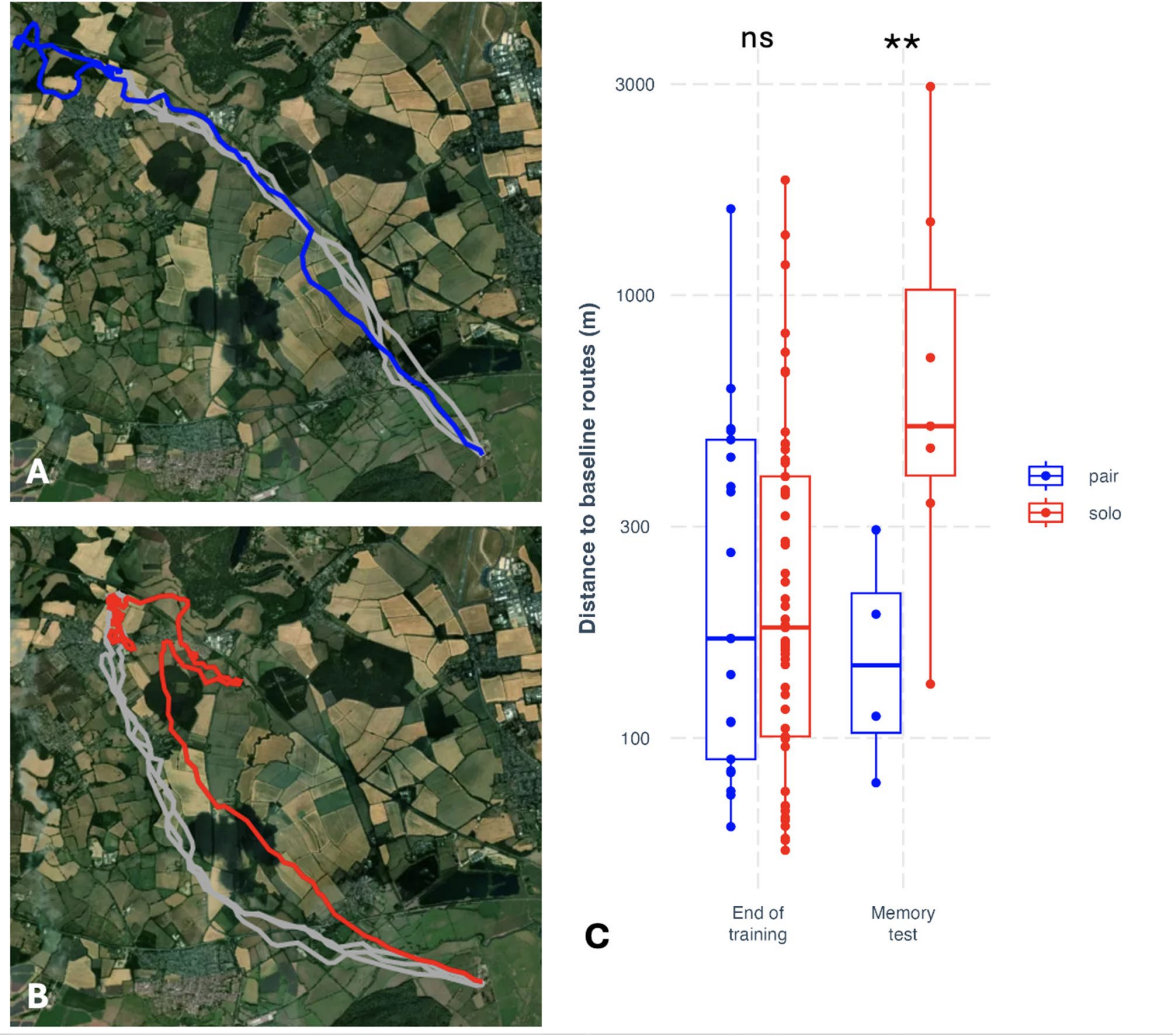
Collective route memories in homing pigeons. (**A**) shows, in grey, the three baseline routes of a pair at the end of training from a release site in the North-West (top-left) of the map to their home loft in the South-East. The same pair’s trajectory in a memory test eight weeks later is shown in blue. The map was generated by the authors using Plotly (Python, version 3.11.7; https://plotly.com/python/) with Mapbox satellite basemap tiles (https://www.mapbox.com/). Map data, Mapbox, OpenStreetMap contributors. (**B**) shows, in grey, the three baseline routes of a pair at the end of training from the same site; the trajectory home of one of the birds when tested solo eight weeks later is shown in red. (**C**) shows route memory in the forgetting treatment, demonstrating the emergence of collective memory in this experiment: the graph shows the mean nearest neighbour distances (mean NND) of pairs and solo-tested birds to any of their baseline routes when tested at the end of training and eight weeks later during memory testing.

In the forgetting treatment, during memory testing, pairs flew closer to their baseline routes from the end of training than solo-tested birds (mean NND: t = 2.63, *p* = 0.009; 2nd order mean NND: t = 3.09, *p* = 0.002; in both cases, full models were fit with 231 observations from 14 pairs / 28 birds; see details in Methods). This appeared to result from forgetting, as there was no difference between paired and solo birds at the end of training in how close they flew to their previous baseline routes (mean NND: t = 1.16, *p* = 0.249; 2nd order mean NND: t = 0.385, *p* = 0.700), and there was a significant interaction between testing time (baseline vs. at memory testing after period of forgetting) and condition (paired vs. solo) of release (mean NND: t = 2.88, *p* = 0.023; 2nd order mean NND: t = 3.02, *p* = 0.003). These results demonstrate the emergence of collective memory in this experiment (Fig. 2), with the collectively learned solution persisting better in groups even as individual memory degrades. During memory testing in this treatment, we obtained solo homing tracks from *both* members of a pair on only one occasion. Nonetheless, we found, depending on the metric used, that pairs were marginally closer (mean NND: t = 1.66, *p* = 0.100; full model fit with 129 observations from 14 pairs / 28 birds; see details in Methods) or significantly closer (2nd order mean NND: t = 2.60, *p* = 0.010; full model fit with 129 observations from 14 pairs / 28 birds; see details in Methods) to their baseline routes than the better individual bird from the pair both tested solo. This suggests that collective memory emerged from independent forgetting across pairs, rather than leadership by the better-performing member, albeit this result should be treated very cautiously, as it relies on a single datapoint in one of the relevant groupings. Further evidence is provided by the limited overlap between the solo birds and the tested pairs in memory performance, as shown in Fig. 2C. The inference that distributed memory emerged through forgetting is supported by showing that this only emerged during the forgetting period: at baseline testing, unlike at memory testing, the best solo individuals within pairs were closer to their previous baseline routes than pairs (mean NND: t = 2.27, *p* = 0.025; 2nd order mean NND: t = 2.28, *p* = 0.025), with a significant interaction between testing time and release condition (mean NND: t = 2.21; *p* = 0.029; 2nd order mean NND: t = 3.11, *p* = 0.002). From this we can suggest that collective membership gain, i.e., a memory performance benefit of group membership, only emerged for both birds within pairs after forgetting, and did not manifest at the end of collective learning. Therefore, distributed memory may have emerged here through independent forgetting across the pair of birds, rather than independent learning during collective actions.

**Fig. 3:**
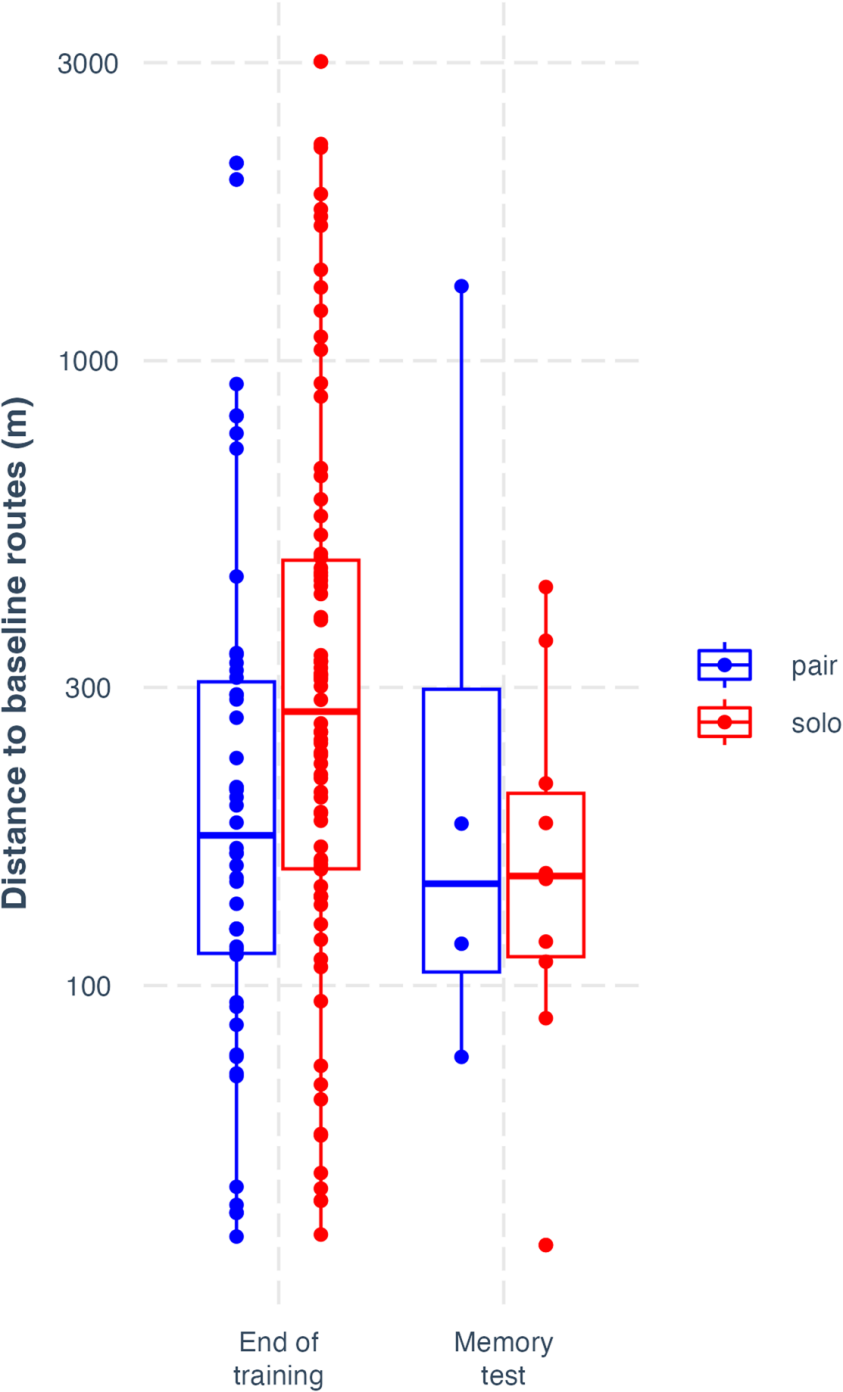
Difference in pair vs. solo performance abolished with extra training treatment. The figure shows the average distances of pairs and solo-tested birds in the extra training treatment to their closest baseline routes (mean NND) when tested at the end of training and at memory testing. The difference between pairs and solo-tested birds in memory testing was abolished by the combination of extra training and a shorter forgetting period.

Conversely, in the extra training treatment, pairs flew no closer than solo-tested pigeons to their baseline routes (mean NND: t = 1.24, *p* = 0.217; 2nd order mean NND: t = 0.47, *p* = 0.636; see Fig. 3). Hence, the combination of extra training and a shorter forgetting period was sufficient to abolish the difference between paired and solo performance in memory tests. This demonstrates that forgetting is necessary for the emergence of a difference between paired and solo birds in memory performance, with additional releases or the timing of memory testing failing to produce a difference between pairs and solo-tested birds in the extra training treatment. Further, in memory testing in the extra training treatment, unlike in the forgetting treatment, pairs did not significantly outperform the better solo-tested birds of pairs both tested solo, instead with better solo-tested birds outperforming pairs in memory testing, depending on the metric, marginally (mean NND: t = 1.91, *p* = 0.059), or non-significantly (2nd order mean NND: t = 0.53, *p* = 0.595). This confirms that no hallmarks of distributed memory emerged in the extra training treatment and demonstrates that the emergence of collective distributed memory was contingent on a longer period of forgetting, rather than other factors such as the timing of memory testing or receiving further releases.

**Fig. 4:**
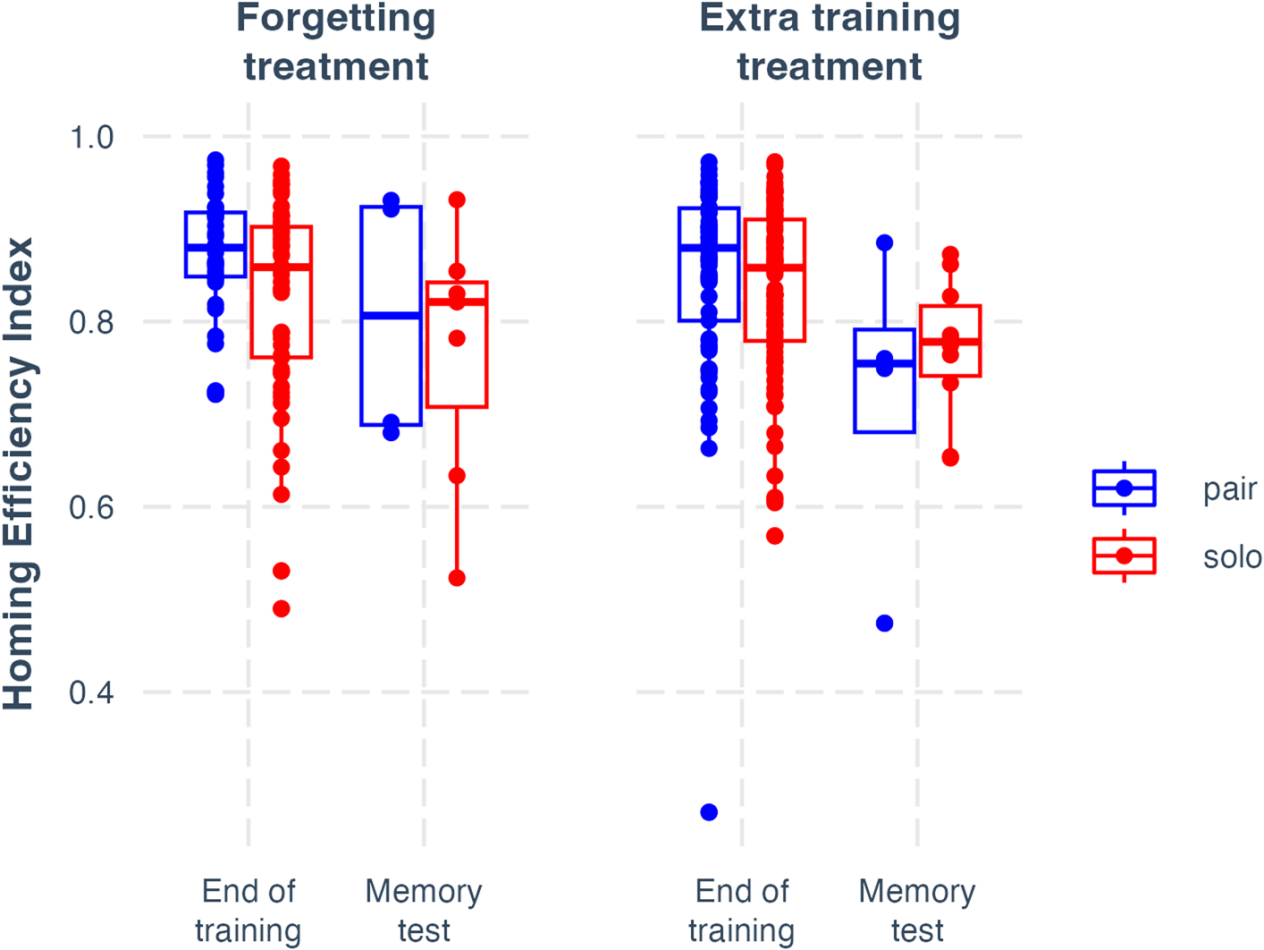
Limited drops in homing efficiency. The homing efficiencies of pairs and solo-tested birds across both treatments are shown. We only found significant drops in homing efficiency between the end of training and memory testing in the extra training treatment, and not the forgetting treatment. No differences between pair and solo efficiencies were detected.

However, the emergence of collective memory in the forgetting treatment did not translate into a difference in the homing efficiency between paired and solo birds, and hence collective intelligence in route efficiency did not emerge in this experiment. Indeed, in both treatments, there was no difference in homing efficiency between paired and solo pigeons after the period of forgetting (forgetting treatment: t = 0.956, *p* = 0.340; extra training treatment: t = 0.543, *p* = 0.588; the full model was fit with 265 observations from 14 pairs / 28 birds; see sample size details in Methods). Moreover, in the forgetting treatment, there was no significant drop in homing efficiency between baseline testing and memory testing, either for paired-tested (t = 1.21; *p* = 0.228) or solo-tested birds (t = 1.69; *p* = 0.093). This contrasts with a previous experiment on homing pigeons^19^ in which partial route forgetting was associated with a drop in homing efficiency. However, that experiment tested memory performance over much longer timescales, comprising several years, rather than the eight-week interval used here. On the other hand, we observed a significant drop in homing efficiency in the extra training treatment between baseline testing and memory testing for both pairs (t = 2.79; *p* = 0.006) and solo-tested birds (t = 2.72; *p* = 0.007). Despite this, there was no significant difference between treatments in their change in homing efficiency from the end of training to memory testing, for either pairs (t = 1.08; *p* = 0.280) or solo-tested birds (t = 0.433; *p* = 0.665). Therefore, we cannot conclude that there was any difference in the changes in homing efficiency from the end of training to memory testing between the treatments. These results are shown in Fig. 4. From these results, we infer that the period of forgetting used in this experiment was insufficient to generate enough forgetting to translate into a drop in homing efficiency across both treatments. This may account for the absence of collective intelligence in this experiment despite the emergence of collective memory.

## Conclusions

Hence, this study provides evidence that collective memory emerges in pairs of co-navigating homing pigeons, likely through independent forgetting across group members generating distributed route memories within pairs. While this appears to have generated independent information across members to contribute to collective decision-making, it did not result in a collective intelligence effect in this experiment. However, this may be explained by the forgetting period being insufficiently long to generate detectable drops in homing efficiency across all treatment groups, and, in particular, in the solo-tested forgetting group. We predict that collective intelligence would emerge after a longer forgetting period that generated larger drops in performance than observed in this experiment. Nonetheless, the emergence of collective memory through independent forgetting across group members highlights an unexplored mechanism through which collective intelligence might manifest in groups with stable membership. Further, we found suggestive evidence that a benefit of group membership in route retention emerged for both members of a pair in this experiment. Again, this emerged as a result of forgetting, with only the worse-performing members of each pair gaining performance benefits from group membership at the end of learning. Whether benefits in route retention would translate into improvements in efficiency over longer time-scales remains an open question; this may emerge through retention of high-efficiency routes, like in previous pigeon experiments^19^. Conversely, it is possible that high-fidelity route memory may constrain improvements in route efficiency through learning, and even that initial drops in efficiency after forgetting may ultimately translate into reaching higher route efficiencies through development and recombination of partially-remembered routes.

There is the potential for collective memory, the better retention of learned information in larger groups, to emerge in various contexts, both spatial and non-spatial, in which learning and memory plays a role in collective behaviour in animals. Further, this may represent an unexplored general mechanism for enhancing group decision-making, generating collective intelligence. Future work should address the interplay of learning and memory in shaping individual and collective behaviour and facilitating the emergence of collective intelligence in animals.

## Methods

### Study system

This study was conducted with a captive population of homing pigeons at the John Krebs Field Station, Oxford, UK (51.7828602, -1.3173753). In March and April of 2024, the pigeons underwent a pretraining phase to familiarise them with the experimental procedures. During this phase, the pigeons were released from four distinct sites, each about 2 km from their home loft, in different compass directions. Each bird was released four times in flocks and four times individually from each site. Additionally, during this period, the pigeons were acclimated to wearing harnesses and carrying plasticine weights (~ 15 g), which represented less than 5% of their body mass and matched the weight of the GPS devices used in the trials. In the experiment, homing trajectories were tracked using GPS devices mounted on the pigeons’ harnesses, which recorded positional data at a 1 Hz resolution (Mobile Action iGot-U GT120). This work was approved by the Animal Welfare and Ethical Review Board of the Department of Biology of the University of Oxford, in accordance with University policy on the use of protected animals for scientific research, and conformed to the relevant regulatory standards.

### Route training

In May and June 2024, pigeons underwent training through successive releases at two sites (Stanton Harcourt site: 51.7527778, -1.42375, 65° degrees and 8.1 km to home; Long Hanbrough site: 51.8303056, -1.3915, 135° degrees and 7.3 km to home). Pigeons received two pre-training releases at each site in flocks of 6 to 8 birds. They were then randomly assigned into pairs and repeatedly released in these pairs at both sites. Once released, pigeons were observed until they disappeared from sight before the next pair were released at least three minutes later.

Pigeons were released in pairs 14 times at each site. The final three paired releases were interspersed with three solo releases and pigeons were GPS-tracked during these six releases.

Pairs were randomly assigned to a treatment at each of the two sites, such that each pair undertook both treatments, one at each site, and such that there were an equal number of pairs undergoing each treatment at each site. The two treatments were as follows:

1. Forgetting treatment: eight-week gap from end of training to memory testing.
2. Extra training treatment: nine additional paired training releases, plus two solo releases, in the three weeks after training and then a five-week gap to memory testing. The last three releases in this extra training were GPS-tracked (two paired and 1 solo).

### Memory testing

For memory testing, pigeons were randomly assigned to either paired or solo releases at each site, constrained such that there were approximately equal numbers of pair and solo birds for both treatments and at both sites. For any pigeons that had to be excluded from the experiment between training and memory testing (in instances of the bird going missing or losing condition), its partner was assigned to the solo release treatment at both sites. Like in training, paired and solo releases were interspersed at each site during memory testing.

### Analysis: route memory and homing efficiency

All GPS tracks were processed to remove stationary points with a speed filter of 30 km/h. Additionally, they were trimmed to include only the homing sections of the tracks, between where the birds left 2 km of the release site for the final time and where the birds entered within 500 m of their home loft.

To identify cases in which the pigeons that had been released separately joined and flew together post-release, the tracks were cross-checked every 30 s for points on other tracks within 50 m. Any instances in which pairs of tracks met this criterion were manually checked by visual inspection on a map with both tracks superimposed. Any tracks that were determined, through visual inspection, to include sections in which the pigeons had joined and flown together were excluded from all analyses. Birds were considered to have split if they moved more than 150 m apart and did not re-establish proximity within that distance. Any sections of paired tracks after the birds had split were excluded from all analyses.

Tracked paired releases at the end of training (and extra training) were used as baseline routes in the analysis. Hence, at the end of training, each pair had up to three baseline routes at each site (fewer if any had to be excluded according to criteria above), and at the end of extra training, each pair had up to five baseline routes at that site. To assess route memory at the end of training and extra training, each track was compared to previously recorded baseline routes; to assess route memory during memory testing, tracks were compared to all baseline routes of that pair. Route memory was quantified in two ways:

1. Mean NND: the mean distance between each point on memory testing track and the closest point on any of the previously recorded baseline tracks for that bird.
2. 2nd order mean NND: the mean distance between each point on the memory testing track and the closest point on each baseline route, averaged across all baseline routes for that bird. Replicating results with this measure ensured that no artefactual results had arisen with the first measure owing to potentially variable number of baseline routes across different comparisons.

Homing efficiency was calculated as the distance between the first and last points on the track divided by the distance travelled, as in, for example^20,21^.

Analysis was performed using a single datapoint per pair or solo bird per release to avoid pseudo-replication, so for data from paired releases, the mean of the measures of the response variable from the tracks of the two birds was used (these two were extremely close to each other in all cases). The data were analysed using multimember linear mixed-effects models with the following formula: Response ~ Group_condition*Treatment*Testing_time + Site + (1 | Individual_ID) + (1 | Pair_ID). Group_condition (levels: paired, solo), Treatment (levels: forgetting treatment, extra training treatment), Testing_time (levels: baseline, memory testing), and Site (levels: A, B) were treated as categorical variables. P-values for fixed effects were obtained using Wald tests, based on model estimates and standard errors.

To assess whether these models met the assumption of normally distributed model residuals, we performed the Kolmogorov-Smirnov test for normality and visually inspected the quantile-quantile plots of the standardised residual quantiles against theoretical quantiles. After these checks, we transformed the response variables: we used natural logarithm transformations of the route memory metrics; for the homing efficiency index (HEI), we used the transformation: y = log((1 – HEI)/HEI), with a natural logarithm. After these transformations, we found that the Kolmogorov-Smirnov test did not find significant deviations from normality of model residuals. These transformations did not substantively influence our findings.

Analyses were conducted using R version 4.3.3 (2024-02-29), with packages geosphere, dplyr, tidyr, circular, lmerMultiMember, lme4, car, jtools, parameters, ggplot2, and emmeans.

### Sample sizes

**Route memory metrics**

**Table:** 

Group	*n* tracks	*n* subjects (pair or solo) ^$^
Forgetting treatment; Pairs; Memory testing	4	4
Forgetting treatment; Solos; Memory testing	7	7
Forgetting treatment; Pairs; End of training	21	14
Forgetting treatment; Solos; End of training	53	26
Extra training; Pairs; Memory testing	4	4
Extra training; Solos; Memory testing	10	10
Extra training; Pairs; End of training	50	14
Extra training; Solos; End of training	82	28

^$^Number of subjects across both sites (a subject may be represented in multiple cells).

**Homing efficiency**.

**Table:** 

Group	*n* tracks	*n* subjects (pair or solo) ^$^
Forgetting treatment; Pairs; Memory testing	4	4
Forgetting treatment; Solos; Memory testing	7	7
Forgetting treatment; Pairs; End of training	35	14
Forgetting treatment; Solos; End of training	53	26
Extra training; Pairs; Memory testing	4	4
Extra training; Solos; Memory testing	10	10
Extra training; Pairs; End of training	65	14
Extra training; Solos; End of training	87	28

^$^Number of subjects across both sites (a subject may be represented in multiple cells).

**Route memory metrics (pair vs. better solo bird of pairs both tested solo)**

**Table:** 

Group	*n* tracks	*n* subjects (pair or solo) ^$^
Forgetting treatment; Pairs; Memory testing	4	4
Forgetting treatment; Solos; Memory testing	1	1
Forgetting treatment; Pairs; End of training	21	14
Forgetting treatment; Solos; End of training	17	15/14 ^+^
Extra training; Pairs; Memory testing	4	4
Extra training; Solos; Memory testing	3	3
Extra training; Pairs; End of training	50	14
Extra training; Solos; End of training	29	21/19 ^+^

^$^Number of subjects across both sites (a subject may be represented in multiple cells).

^+^Different number of subjects for the two metrics: mean NND / 2nd order mean NND.

## Data Availability

The code and data used in this project can be found here: https://github.com/Morfordjoe/Collective-route-memories-in-homing-pigeons.

## References

[1] Krause, J., Ruxton, G. D. & Krause, S. Swarm intelligence in animals and humans. *Trends Ecol. Evol.* **25** (1), 28–34. 10.1016/j.tree.2009.06.016 (2010). 19735961 10.1016/j.tree.2009.06.016

[2] Simons, A. M. Many wrongs: the advantage of group navigation. *Trends Ecol. Evol.* **19** (9), 453–455. 10.1016/j.tree.2004.07.001 (2004). 16701304 10.1016/j.tree.2004.07.001

[3] Condorcet, M. d. Essay on the Application of Analysis to the Probability of Majority Decisions. *Paris: Imprimerie Royale*, 1785. (1785).

[4] Biro, D., Sasaki, T. & Portugal, S. J. Bringing a Time-Depth perspective to collective animal behaviour. *Trends Ecol. Evol.* **31** (7), 550–562. 10.1016/j.tree.2016.03.018 (2016). 27105543 10.1016/j.tree.2016.03.018

[5] Hinsz, V. B. Cognitive and consensus processes in group recognition memory performance. *J. Personal. Soc. Psychol.* **59** (4), 705 (1990).

[6] Weldon, M. S. & Bellinger, K. D. Collective memory: collaborative and individual processes in remembering. *J. Exp. Psychol. Learn. Mem. Cogn.* **23** (5), 1160–1175. https://doi.org/10.1037//0278-7393.23.5.1160 (1997). 10.1037//0278-7393.23.5.1160 9293627

[7] Wilson, R. A. Collective memory, group Minds, and the extended Mind thesis. *Cogn. Process.* **6** (4), 227–236. 10.1007/s10339-005-0012-z (2005). 18239951 10.1007/s10339-005-0012-z

[8] Morford, J. et al. Neural networks reveal emergent properties of collective learning in Democratic but not despotic groups. *Anim. Behav.* **194**, 151–159. 10.1016/j.anbehav.2022.09.020 (2022). 41357475 10.1016/j.anbehav.2022.09.020 PMC7618439

[9] Kao, A. B. & Couzin, I. D. Decision accuracy in complex environments is often maximized by small group sizes. *Proceedings of the Royal Society B: Biological Sciences*. **281**(1784), 20133305 10.1098/rspb.2013.3305 (2014). 10.1098/rspb.2013.3305 PMC4043084 24759858

[10] Kao, A. B., Miller, N., Torney, C., Hartnett, A. & Couzin, I. D. Collective learning and optimal consensus decisions in social animal groups. *PLoS Comput. Biol.* **10** (8), e1003762. 10.1371/journal.pcbi.1003762 (2014). 25101642 10.1371/journal.pcbi.1003762 PMC4125046

[11] Surowiecki, J. *The wisdom of crowds: why the many are smarter than the few and how collective wisdom shapes business, economies, societies, and nations* (1st ed.). Doubleday:. Contributor biographical information (2004). http://www.loc.gov/catdir/bios/random055/2003070095.html

[12] Webster, M. M., Whalen, A. & Laland, K. N. Fish pool their experience to solve problems collectively. *Nat. Ecol. Evol.* **1** (5), 0135. 10.1038/s41559-017-0135 (2017). 10.1038/s41559-017-0135 28812697

[13] Sasaki, T., Masuda, N., Mann, R. P. & Biro, D. Empirical test of the many-wrongs hypothesis reveals weighted averaging of individual routes in pigeon flocks. *Iscience*, **25** (10), 105076 (2022). 10.1016/j.isci.2022.105076 PMC9485075 36147962

[14] Collet, J. et al. Mechanisms of collective learning: how can animal groups improve collective performance when repeating a task? *Philosophical Trans. Royal Soc. B: Biol. Sci.* **378** (1874), 20220060. 10.1098/rstb.2022.0060 (2023). 10.1098/rstb.2022.0060 PMC9939276 36802785

[15] Flack, A., Freeman, R., Guilford, T. & Biro, D. Pairs of pigeons act as behavioural units during route learning and co-navigational leadership conflicts. *J. Exp. Biol.* **216** (Pt 8), 1434–1438. 10.1242/jeb.082800 (2013). 23536590 10.1242/jeb.082800

[16] Sasaki, T. & Biro, D. Cumulative culture can emerge from collective intelligence in animal groups. *Nat. Commun.* **8**, 15049. 10.1038/ncomms15049 (2017). 28416804 10.1038/ncomms15049 PMC5399285

[17] Biro, D., Meade, J. & Guilford, T. Familiar route loyalty implies visual pilotage in the homing pigeon. *Proc. Natl. Acad. Sci.* **101** (50), 17440–17443 (2004). 15572457 10.1073/pnas.0406984101 PMC536010

[18] Meade, J., Biro, D. & Guilford, T. Homing pigeons develop local route stereotypy. *Proc. Royal Soc. B: Biol. Sci.* **272** (1558), 17–23 (2005). 10.1098/rspb.2004.2873 PMC1634935 15875565

[19] Collet, J., Sasaki, T. & Biro, D. Pigeons retain partial memories of homing paths years after learning them individually, collectively or culturally. *Proc. Royal Soc. B*. **288** (1963), 20212110 (2021). 10.1098/rspb.2021.2110 PMC8595992 34784759

[20] Gagliardo, A., Pollonara, E. & Wikelski, M. Pigeon navigation: exposure to environmental odours prior to release is sufficient for homeward orientation, but not for homing. *J. Exp. Biol.* **219** (Pt 16), 2475–2480. 10.1242/jeb.140889 (2016). 27284069 10.1242/jeb.140889

[21] Morford, J., Gagliardo, A., Pollonara, E. & Guilford, T. Homing pigeon navigational ontogeny: no evidence that exposure to a novel release site is sufficient for learning. *Anim. Behav.* **214**, 157–164. 10.1016/j.anbehav.2024.06.009 (2024). 39469529 10.1016/j.anbehav.2024.06.009 PMC11512678

